# Comparison of QM Methods for the Evaluation of Halogen−π Interactions for Large-Scale Data Generation

**DOI:** 10.1021/acs.jctc.5c00456

**Published:** 2025-06-09

**Authors:** Marc U. Engelhardt, Markus O. Zimmermann, Finn Mier, Frank M. Boeckler

**Affiliations:** † Laboratory for Molecular Design & Pharmaceutical Biophysics, Institute of Pharmaceutical Sciences, Department of Pharmacy and Biochemistry, Eberhard Karls Universität Tübingen, 72076 Tübingen, Germany; ‡ Interfaculty Institute for Biomedical Informatics (IBMI), Eberhard Karls Universität Tübingen, 72076 Tübingen, Germany

## Abstract

Halogen−π interactions play a pivotal role in molecular recognition processes, drug design, and therapeutic strategies, providing unique opportunities for enhancing and fine-tuning the binding affinity and specificity of pharmaceutical agents. The present study systematically benchmarks various combinations of quantum mechanical (QM) methods and basis sets to characterize halogen−π interactions in model systems. We evaluate both density functional theory (DFT) methods and wave function-based post-HF methods in terms of accuracy to reference calculations at the CCSD­(T)/CBS level of theory and runtime efficiency. By balancing these crucial aspects, we aim to identify an optimal configuration suitable for high-throughput applications. Our results indicate that MP2 using the reasonably large TZVPP basis set is in excellent agreement with reference calculations, striking a balance between accuracy and computational efficiency. This allows us to generate large, reliable data sets, which will serve as a basis to develop and train machine-learning models capable of accurately capturing the strength of halogen−π interactions, thereby providing a robust data-driven foundation for medicinal chemistry analysis.

## Graphical abstract

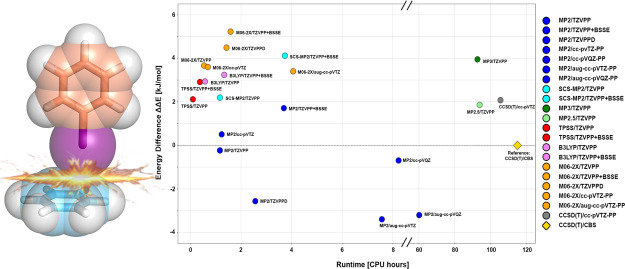

## Introduction

Noncovalent interactions play a fundamental role in biological systems and molecular recognition processes, serving as the keystone for understanding biomolecular function, drug binding, and protein–ligand interactions.^1–5^ Among these, halogen bonding (XB) has emerged as a unique and versatile interaction, characterized by the directional attraction between an electrophilic region on a halogen atom (σ-hole), typically chlorine, bromine, or iodine, and a nucleophilic partner.^6–12^ These interactions have proven particularly useful in medicinal chemistry and drug design, where they not only enhance the binding affinity and specificity of ligands and stability of protein–ligand complexes^13–21^ but also can contribute to ligands engaging in unconventional binding modes.^22–24^

Various nucleophilic moieties that form noncovalent interactions with a halogen atom in the protein binding site have been systematically investigated.^25–27^ Although XB acceptors such as backbone carbonyls and the π-surface of the peptide bond,^28,29^ the sulfur atom in methionine,^30^ the nitrogen atoms in histidine,^31^ carboxylate (aspartate/glutamate) and carboxamide (asparagine/glutamine) moieties,^32^ and oxygen atoms of water molecules^33,34^ have already been studied extensively, the importance of addressing π-systems (aromatic side chains of tyrosine, phenylalanine, histidine, and tryptophan) as XB acceptors in protein–ligand interactions has only been highlighted and systematic approaches are still underrepresented.^35–38^

To date, the nature of halogen bonding is still a controversial subject, and thus, theoretical calculations are of tremendous importance.^39–43^ In 2012, Rezac et al.^44^ analyzed and benchmarked calculations of various noncovalent interactions of halogenated molecules using different quantum mechanical (QM) methods and basis sets and generated a small benchmark set of interaction geometries. With respect to halogen−π interactions, halomethane or a tuned variant, trifluorohalomethane, in complex with a benzene molecule, was used. In 2020, Zhu et al.^45^ published a perspective on the application of QM methods to evaluate halogen bonding, where they further investigated halogen interactions in general, including aromatic systems as acceptors. Despite their initial and pioneering work, in-depth analysis on a larger scale, especially for halogen bonding donors and acceptors with relevance to the drug discovery process, is still limited. Accurate modeling of these interactions is essential for capturing their energetic contributions to protein–ligand binding and their application in guiding structure-based drug design. Wallnoefer et al.^38^ investigated interactions of chlorobenzene and bromobenzene, addressing a *p*-cresol system, and provided an initial comparison of QM methods and basis sets for such systems.

QM methods differ in how they treat electronic interactions, electron correlation, and exchange effects, impacting their accuracy and computational cost.^46^ Balancing these factors is crucial, especially when dealing with complex biological systems. Among several methods, the coupled cluster method CCSD­(T) is widely used as the “gold standard” because it is the most accurate, nonempirical method applicable to reasonably large systems of practical interest.^47^ Mo̷ller-Plesset perturbation theory (MPn)^48^ methods are post-Hartree–Fock approaches that explicitly account for electron correlation by systematically improving the wave function obtained from the Hartree–Fock calculation using *n*th order perturbation theory. MP2 ^49^ (second-order Mo̷ller-Plesset) is widely used for its balance between accuracy and computational cost. MP3 ^50,51^ extends this approach by including third-order terms, offering improved accuracy but at an exponentially higher computational cost. MP2.5 is a pragmatic compromise that averages MP2 and MP3 energies, often providing results closer to CCSD­(T) with reduced computational requirements in comparison to coupled cluster calculations.^52^ This approach exploits the systematic error compensation between MP2’s tendency to underestimate and MP3′s tendency to overestimate in some systems. Spin-Component-Scaled MP2 (SCS-MP2 ^53,54^ ) refines MP2 by applying different scaling factors to the parallel spin and opposite spin electron correlation components. This adjustment improves the accuracy by reducing the tendency to overestimate electron correlation effects by MP2.

In contrast, density functional theory methods (DFT) approximate the electron correlation through exchange-correlation functionals based on electron density rather than the wave function.^55^ TPSS^56^ is a GGA^57^ (generalized gradient approximation) functional that includes a kinetic energy density term, improving the accuracy for weak interactions and transition metal chemistry. B3LYP^58,59^ and M06–2X^60^ are popular hybrid GGA functionals, mixing parts of the exact Hartree–Fock exchange with DFT exchange-correlation functionals, to balance computational cost and accuracy. Accuracy of the DFT methods can be enhanced by adding Grimme’s D3 dispersion correction for noncovalent interactions.^61^

Besides the choice of an appropriate QM method, a reasonable choice of the basis set for the calculation is certainly important. Commonly used basis sets include the triple-ζ valence with polarization (TZVPP^62^) or an enhanced variant with an additional diffuse function (TZVPPD), the correlation-consistent polarized valence X-ζ^63^ (cc-pVXZ, where X = *D*, *T*, *Q*, etc.), and its extended counterpart, aug-cc-pVXZ.^64^ The TZVPP basis set, widely used in density functional theory (DFT) and post-Hartree–Fock methods, provides a robust trade-off between computational efficiency and accuracy by including multiple polarization functions. The cc-pVXZ family of basis sets, on the other hand, is designed to improve electron correlation effects with increasing cardinality X. The extended versions (aug-cc-pVXZ) also include diffuse functions, which further enhance contributions of dispersion effects for noncovalent interactions. The suffix “-PP” indicates an additional pseudopotential for certain higher-order atoms, such as iodine. Although such calculations are practically impossible, the most accurate result would be achieved by CCSD­(T) calculations using a complete basis set (CBS). To address this issue, calculations using smaller basis sets, typically correlation-consistent basis sets, can be extrapolated to the complete basis set limit.^65^

In this study, we focus on the systematic investigation of halogen−π interactions using high-level quantum mechanical (QM) methods, including post-Hartree–Fock and DFT, in combination with commonly used basis sets. We aim to select a proper method with a reasonable balance between speed and accuracy, in order to apply this method for generating big data sets of several million halogen−π interaction geometries. Based on this big data, we will strive to derive models from machine learning approaches that enable us to predict the interaction energy for a given geometry almost instantly without the need for calculations at the QM level.

A systematic grid of iodobenzene as a ligand model system in complex with benzene as a halogen bond acceptor is generated, and single-point calculations are carried out. Our focus clearly is on iodobenzene, as a complementary halogen bond donor to previous studies,^38^ but also, particularly, because it provides the strongest halogen bonds in comparison to bromobenzene or chlorobenzene. To ensure comparability and applicability among the halobenzenes, single-point calculations for combinations of methods and basis sets of particular importance were conducted for chlorobenzene and bromobenzene as well. Calculations on the CCSD­(T) level of theory extrapolated to the complete basis set limit serve as a reference. We report energy differences and computational costs (CPU runtime) to evaluate the most suitable method and basis set combination for representing halogen−π interactions. From previous studies,^27,28,30–32^ we have often observed good applicability of calculations on an MP2/TZVPP level of theory. Thus, we were interested to see whether this experience could be transferred to noncovalent interactions with π-systems.

## Results and Discussion

### Comparison of QM Methods and Basis Sets

In comparison to interactions involving single atom acceptors, such as oxygen or nitrogen, halogen−π interactions in haloaryl systems (where the halogen atom is directly attached to an aromatic ring) exhibit greater structural diversity. This is based on the strong increase of possible interaction geometries, correlating to the larger π-surface for forming attractive interactions. This difference arises from the extended π-plane of the aromatic systems, which provides a delocalized electron cloud capable of interacting with the σ-hole of the halogen atom in multiple geometric orientations. In contrast, the localized lone pair electrons of oxygen or nitrogen constrain the halogen bond donors to fewer but more specific orientation geometries, showing less flexibility than the π-systems. Still, the extended π-surface is also a “double-edged sword”, as it increases the risk for the formation of secondary interactions such as π···π or C–H···π.

Researchers have reported different results and opinions regarding the suitability of different QM methods for halogen bonding. However, our group’s previous investigations at the MP2 level, using a triple-ζ (def2-TZVPP) basis set on different halogen bond acceptors, yielded accurate adduct formation energies while maintaining a feasible computational time. To ensure that this level of calculation is also suitable for halogen−π interactions, we conducted benchmark calculations of different combinations of QM methods and basis sets. Adduct formation energies were compared to high-level reference calculations on a CCSD­(T) level of theory, extrapolated to the basis set limit using the approach proposed by Halkier et al.^65^ It should be noted that we will use the short-term “CCSD­(T)/CBS” subsequently as always referring to this complete basis set extrapolation approach. At this level of theory, only a smaller subset (∼30% of all geometries) was used due to the extraordinarily high computational cost. Several applied methods were counterpoise corrected (BSSE correction) with the procedure of Boys and Bernardi.^66^ However, it has to be noted that the effectiveness of BSSE correction remains a controversial subject in the literature.^67^

Table 1 shows the mean energy deviations (ΔΔ*E*), the mean absolute energy deviations (|ΔΔ*E*|), and the root-mean-square deviations (RMSD) in kJ/mol from the reference CCSD­(T)/CBS, together with the corresponding computational cost in CPU hours. A detailed table incorporating the individual data points and energies of all methods is provided as a separate Excel file, which can be found as part of the Supporting Information. Mean differences, calculated as ΔΔ*E* = Δ*E*_method_–Δ*E*_CCSD(T)/CBS_, where Δ*E* denotes the adduct formation energy, indicate an overall deviation between the methods and their tendency to over- or underestimate energies. However, these values can be misleading, as large positive and negative values can level each other out. Therefore, we additionally report the mean absolute energy deviation and the RMSD. RMSD values can give further insight into the magnitude of the large difference compared to |ΔΔ*E*|. The runtime is averaged over single-points of different distances and across the calculated grid. It is obvious that the CCSD­(T) reference calculations require by far the highest resources, with about 115 h per single-point. The majority of the computational costs for extrapolation are caused by the CCSD­(T)/cc-pVTZ-PP calculation (105.51 h).

**Table 1:** Mean Energy Difference, ΔΔ*E*, Mean Absolute Energy Differences (|ΔΔ*E*|), and RMSD of Different QM Methods and the Used Basis Sets^b^

method	basis set	mean ΔΔ*E* to CCSD(T)/CBS	abs. mean |ΔΔ*E*| to CCSD(T)/CBS	RMSD to CCSD(T)/CBS	mean runtime (CPUh)
MP2	TZVPP	–0.23	0.64	0.91	1.16
	TZVPP+BSSE	1.71	1.71	2.43	3.69
	TZVPPD	–2.56	2.57	3.51	2.56
	cc-pVTZ-PP	0.51	0.59	0.73	1.23
	cc-pVQZ-PP	–0.69	0.96	1.66	8.23
	aug-cc-pVTZ-PP	–3.39	3.39	4.44	7.57
	aug-cc-pVQZ-PP	–3.20	3.20	4.39	60.14
SCS-MP2	TZVPP	2.19	2.19	3.23	1.16
	TZVPP+BSSE	4.12	4.11	6.18	3.73
MP3	TZVPP	3.95	3.95	6.22	92.68
MP2.5	TZVPP	1.86	1.85	2.83	93.84
TPSS (-D3)	TZVPP	2.12	2.11	4.25	0.09
	TZVPP+BSSE	2.91	2.91	4.81	0.37
B3LYP (-D3)	TZVPP	2.94	2.93	6.25	0.57
	TZVPP+BSSE	3.24	3.23	6.53	1.33
M06–2X (-D3)	TZVPP	3.67	3.66	5.10	0.53
	TZVPP+BSSE	5.23	5.21	6.57	1.58
	TZVPPD	4.49	4.51	11.44	1.42
	cc-pVTZ-PP	3.60	3.59	5.10	0.68
	aug-cc-pVTZ-PP	3.40	3.40	4.86	4.06
CCSD(T)	cc-pVTZ-PP	2.08	2.08	3.46	105.51
CCSD(T)	CBS	^a^	^a^	^a^	114.97

a No values reported, since CCSD­(T)/CBS is the reference for all other methods.

b Energy values are given as difference between corresponding method values and the reference calculations of CCSD­(T)/CBS in kJ/mol. Runtime is given in CPU hours.

Among the evaluated methods, MP2 using the TZVPP basis set stands out with an excellent balance between accuracy and computational cost (Figure 1). The method shows mean deviations and absolute mean deviations of ΔΔ*E* = −0.23 kJ/mol and |ΔΔ*E*| = 0.64 kJ/mol, respectively. This level of accuracy is achieved with a notably low computational cost of about 1.16 CPU hours on average per single-point calculation. In contrast, the BSSE-corrected version of MP2/TZVPP results in larger deviations (ΔΔ*E* = |ΔΔ*E*| = 1.71 kJ/mol, RMSD = 2.43 kJ/mol) and requires significantly more time with 2.56 CPU h, due to the calculations of ghost molecules to eliminate overlapping terms. Calculations using the diffuse function enhanced TZVPPD basis set, unfortunately, show the worst results of the triple-ζ variants with ΔΔ*E* = −2.56 kJ/mol, |ΔΔ*E*| = 2.57 kJ/mol, and RMSD = 3.51 kJ/mol. The correlation-consistent basis sets cc-pVTZ-PP and cc-pVQZ-PP also show very good results with ΔΔ*E* = 0.59 kJ/mol (RMSD = 0.73 kJ/mol) and ΔΔ*E* = 0.96 kJ/mol (RMSD = 1.66 kJ/mol), respectively. However, looking at the runtime, cc-pVTZ-PP with 1.23 CPU hours may still compete with TZVPP, while the larger cc-pVQZ-PP basis set with 8.23 CPU hours on average seems neither efficient nor most effective. Furthermore, augmented basis sets aug-cc-pVTZ-PP and aug-cc-pVQZ-PP both perform quite similarly for all energy results, but the deviations here are rather large and with much higher runtimes of 7.57 and even over 60 CPU hours, respectively.

**Figure 1:**
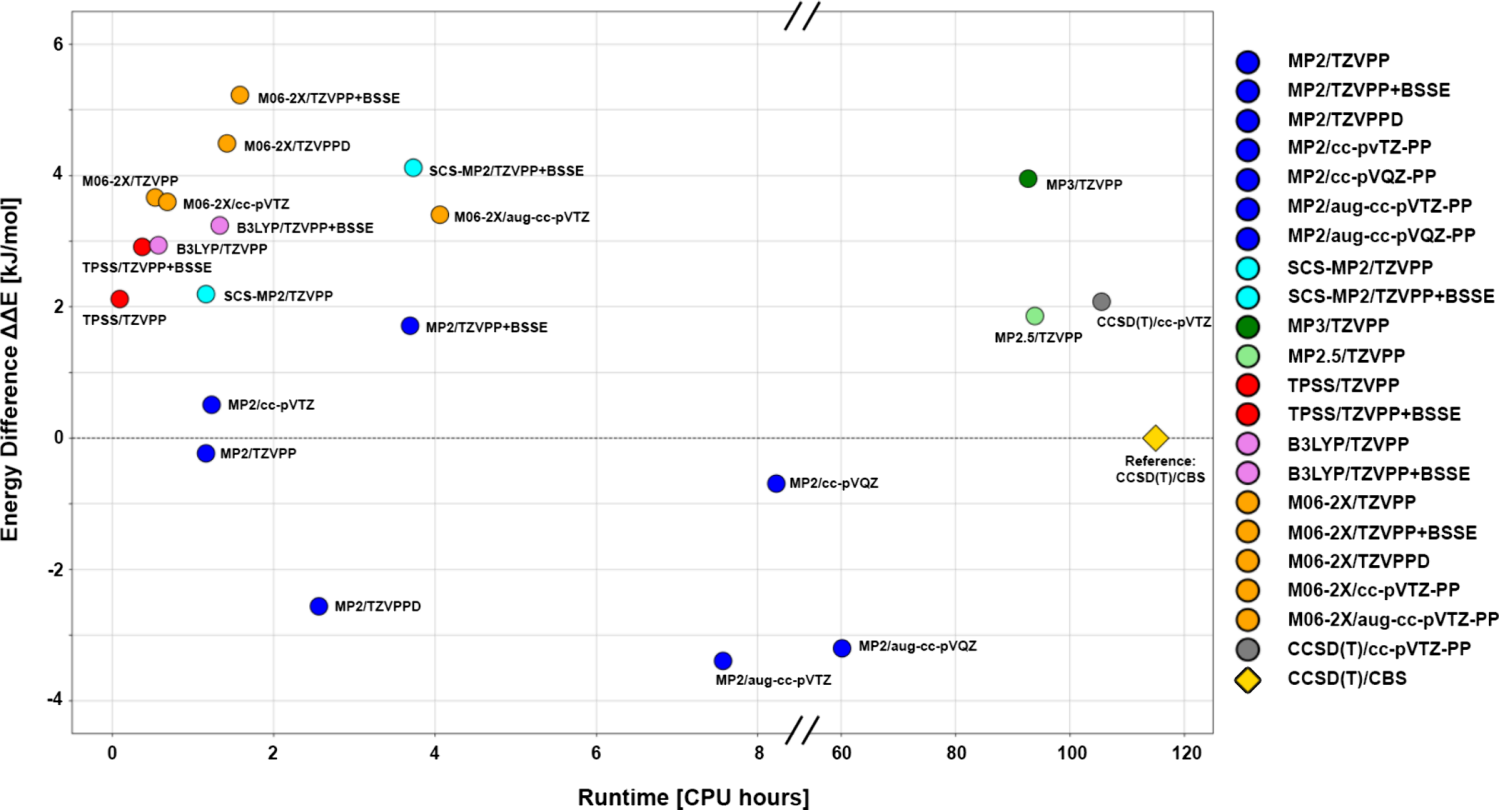
Mean energy difference ΔΔ*E* (in kJ/mol) to the reference CCSD­(T)/CBS and runtime (in CPU hours) of the evaluated methods and basis sets of iodine. The dashed horizontal line at ΔΔ*E* = 0 kJ/mol indicates the CCSD­(T)/CBS reference level (golden diamond), while the *x*-axis break indicates the large jump in computational cost for higher-level methods. Each color corresponds to a different level of theory and basis set treatment as shown in the legend. The figure was prepared by using custom Python scripts and the *matplotlib* library.

As an alternative post-Hartree–Fock method, MP3 should give relatively accurate predictions with slightly underestimated energy values.^68^ With an energy difference of |ΔΔ*E*| = 3.95 kJ/mol (RMSD = 6.22 kJ/mol) and the vast computational effort requiring almost 93 CPU hours, however, it is less practical for routine calculations. Although MP2.5, as the arithmetic mean of MP2 and MP3, should compensate for over- and underestimations of both methods respectively, it shows higher deviations (|ΔΔ*E*| = 1.85 kJ/mol, RMSD = 2.83 kJ/mol) with the runtime obviously dominated by the MP3 calculations.^69^ However, calculation of MP2.5 energies is, of course, cheap if both MP2 and MP3 calculations are conducted.

Computationally less demanding DFT methods, including B3LYP­(-D3) and TPSS­(-D3), generally show larger absolute deviations in this comparison (|ΔΔ*E*| = 2.9 and 2.1 kJ/mol, respectively). Incorporating BSSE correction even increases the difference in energy values. In previous studies, the widely used M06–2X functional showed very accurate results, especially for weak interactions with dispersion contribution (including halogen bonding).^70,71^ Therefore, the comparison between M06–2X and MP2 across different basis sets is of high interest. Although computationally efficient, with runtimes ranging from 0.53 to 4 h depending on the basis set, M06–2X generally shows larger deviations from the CCSD­(T)/CBS reference than MP2. For example, M06–2X/TZVPP shows an absolute deviation of 3.66 kJ/mol (RMSD = 5.1 kJ/mol), and the BSSE-corrected version further increases this deviation to 5.21 kJ/mol (RMSD = 6.57 kJ/mol). This indicates that M06–2X may be suitable for highlighting tendencies but lacks the necessary quantitative accuracy for this application. M06–2X/TZVPPD shows trends similar to those of MP2/TZVPPD in terms of increasing the difference even further. M06–2X/TZVPPD even shows the highest RMSD among all of the tested methods. Using cc-pVTZ-PP and aug-cc-pVTZ-PP yields similarly inaccurate results as MP2 using the same basis sets.

“2D energy surface plots” of the actual adduct formation energies Δ*E* were generated for each of the five different distances individually to highlight attractive and repulsive areas. This means that the surface in plane with the aromatic ring system of benzene is colored at the position of the halogen atom above this plane based on the Δ*E* value for this halogen−π interaction, followed by interpolating between these energies. Furthermore, we generated 2D surface plots of ΔΔ*E*, as well, showing the deviation from the reference calculations of ΔΔ*E* in a similar fashion. For simplicity, here we only compare MP2/TZVPP and M06–2X/TZVPP. Surface plots of Δ*E* and ΔΔ*E* of the remaining methods can be found in the Supporting Information Figures S1 and S2. Figure 2a shows the adduct formation energy surface plots of MP2/TZVPP and M06–2X/TZVPP for all investigated distances (2.75 3.25, 3.50, 3.75, and 4.25 Å) individually as well as the surfaces of the reference CCSD­(T)/CBS energies. For MP2/TZVPP, adduct formation energies Δ*E* range from −18.88 kJ/mol as the most favorable interaction to 31.19 kJ/mol as highly repulsive. Use of M06–2X/TZVPP provides ranges from −11.59 to 53.55 kJ/mol, while “gold standard” CCSD­(T)/CBS yields a range of adduct formation energies from −16.17 to 33.43 kJ/mol. For better visibility, positive energy values were capped at 10 kJ/mol. For *d* = 2.75 Å, mainly repulsive or only minimal attractive interactions can be observed. Increasing the distance rapidly shifts the interaction from repulsive to attractive. Most favorable interactions with minimum energy values can be observed at *d* = 3.5 Å for both methods. Figure 2b shows the energy surface based on the adduct formation energy difference ΔΔ*E* between the two methods and CCSD­(T)/CBS for all distances. Positive and negative values were capped at 10 and −10 kJ/mol, respectively. Original values are provided in spreadsheet format (xlsx) in the Supporting Information. MP2/TZVPP shows very low differences to the reference calculation, ranging from ΔΔ*E* = 0.59 to −2.9 kJ/mol, while for M06–2X/TZVPP, deviations from the reference energies range from ΔΔ*E* = 0.58 to 20.11 kJ/mol. It can be argued that precise predictions of highly repulsive energies are less relevant for drug discovery purposes as long as the strong repulsion is recognized and the area of the transition between attractive and repulsive interactions is not strongly altered. Thus, for benchmarking purposes, we keep them in the data set.

**Figure 2:**
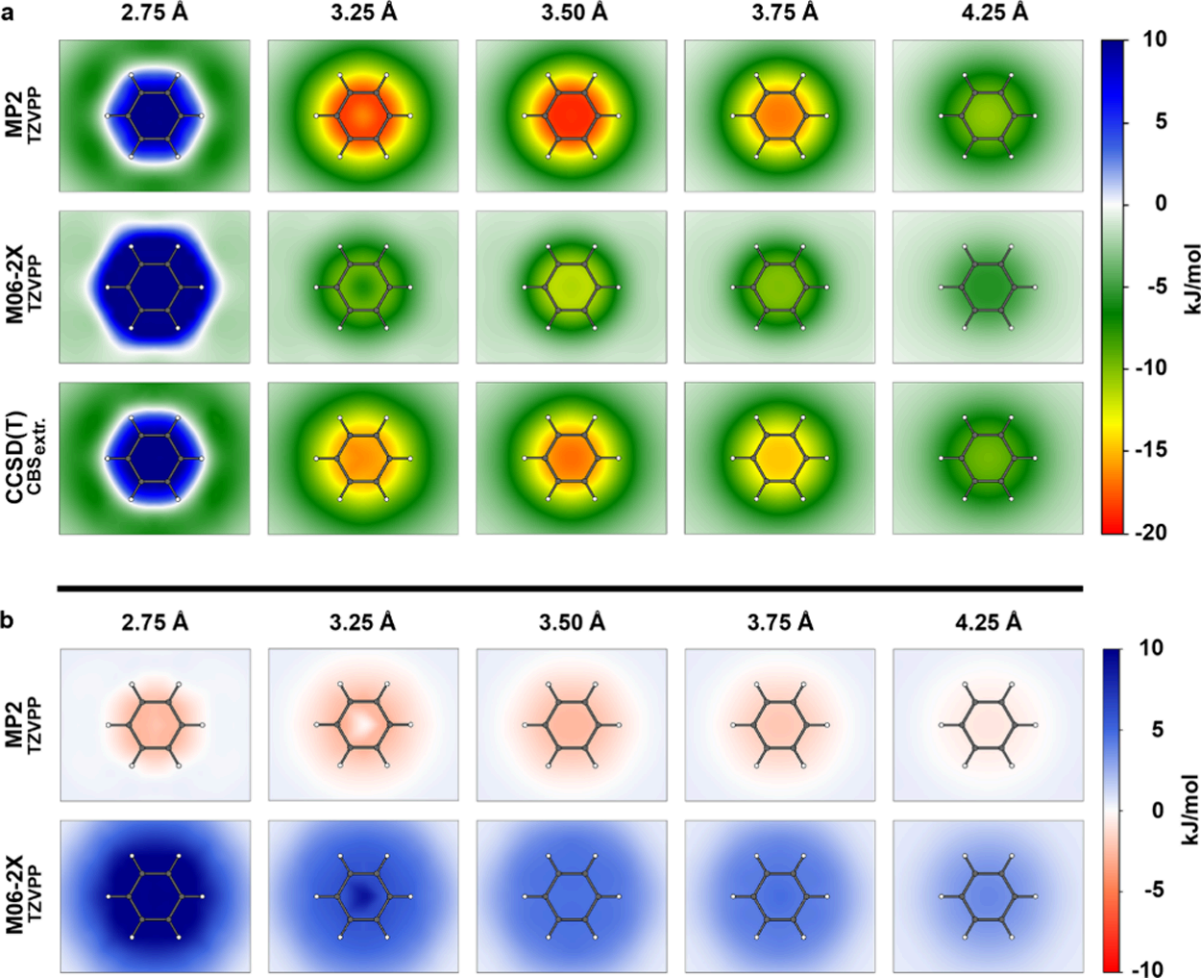
Adduct formation energy surfaces of MP2 and M06–2X with the TZVPP basis set, as well as the surfaces of the reference CCSD­(T)/CBS. Surfaces represent the halogen−π interaction energies Δ*E* of iodobenzene in complex with the targeted benzene at distances of *d*_I···π‑plane_ = [2.75, 3.25, 3.50, 3.75, 4.25 Å]. The iodobenzene is oriented perpendicular to the π-plane. Data points of the surface are interpolated and colored according to the given energy scale. (a) Surfaces of adduct formation energies Δ*E*. Positive energies and negative energies are capped to 10 and −20 kJ/mol, respectively, for better visibility. (b) Surfaces of the difference between adduct formation energies of MP2 and M06–2X and the reference CCSD­(T)/CBS (calculated as ΔΔ*E* = Δ*E*_method_–Δ*E*_CCSD(T)/CBS_). Positive and negative differences were capped to 10 and −10 kJ/mol. Figures were prepared by using custom Python scripts and the *matplotlib* library.

In summary, we find MP2 to be in very good agreement with the “gold standard” CCSD­(T)/CBS across multiple basis sets, with absolute deviations as low as 0.64 kJ/mol for MP2/TZVPP and 0.59 kJ/mol for MP2/cc-pVTZ-PP. RMSD values also show minimal differences to the reference with 0.91 and 0.73 kJ/mol, respectively, with cc-pVTZ-PP performing slightly better. Thus, MP2, using either TZVPP or cc-pVTZ-PP, having almost identical levels of accuracy while maintaining feasible computational effort, seems an excellent choice. Given the overall results and the shorter runtime of MP2/TZVPP (1.16 h) compared to MP2/cc-pVTZ-PP (1.23 h), which could amount to saving several million CPU hours for large data sets, it was emphasized that our previous choice of MP2/TZVPP is a quite reasonable approach.

### Additional Calculations with Chlorobenzene and Bromobenzene

Since interactions of chlorobenzene and bromobenzene with π-systems have been studied previously,^38,44,45^ the focus of this study mainly lies on iodine interactions. Iodine has emerged as a particularly interesting element in medicinal chemistry because its large σ-hole enables the formation of exceptionally strong and highly directional halogen bonds, which medicinal chemists can exploit to modulate the binding affinity, target selectivity, and physico-chemical properties of drug candidates. Although their halogen bonding ability is weaker than iodine’s, traditionally bromine and chlorine remain more prevalent due to their milder steric impact and favorable synthetic versatility. In computational studies, iodine is typically modeled with a relativistic effective-core potential to account for its heavy-atom inner electrons. When benchmarked, conclusions gained for iodine can be confidently extended to its lighter halogen colleagues, bromine and chlorine, whose smaller relativistic contributions arise from the same underlying interactions. To ensure comparability and applicability, single-point calculations of chlorobenzene and bromobenzene in complex with benzene were performed at the MP2 and M06–2X levels of theory using the basis set TZVPP, as well as CCSD­(T)/CBS extrapolation as a reference. The same set of 150 geometries was used for this comparison, applying a proper shift of the halobenzene scaffold to keep the halogen-π distance always identical to the iodine data set. Table 2 shows the results of both chlorine and bromine interactions. Similar to iodine, we report mean energy deviations (ΔΔ*E*), mean absolute energy deviations (|ΔΔ*E*|), and root-mean-square deviations (RMSD) in kJ/mol from the reference CCSD­(T)/CBS, together with the corresponding computational cost in CPU hours. A detailed table incorporating the individual data points and energies of all methods for both chlorine and bromine results can be found in the Supporting Information. It can be concluded that chlorine and bromine interactions behave similarly to those of iodine. Using MP2 with TZVPP yields comparably good results (agreement with reference calculations) for chlorine and bromine as for iodine, while maintaining very low computational costs of around 1–1.3 CPU hours. Interestingly, however, the application of the counterpoise correction differs for chlorine and bromine interactions and shows even better results with lower energy differences from the reference. Unfortunately, MP2/TZVPP+BSSE still shows runtimes of more than 3-fold compared to MP2/TZVPP and thus appears less applicable to the calculation of very large data sets. Looking at the results of M06–2X­(-D3) calculations, trends similar to those of iodine can be derived. While showing rather low computational costs, the energy differences are doubled compared to MP2 calculations. Using the same visualization strategy as in Figure 2, we generated individual “2D energy surface plots” and energy difference plots for each method at every examined distance (2.75, 3.25, 3.50, 3.75, and 4.25 Å) which can be found in the Supporting Information (Figure S3 for chlorine and Figure S4 for bromine).

**Table 2:** Mean Energy Difference, ΔΔ*E*, Mean Absolute Energy Differences (|ΔΔ*E*|), and RMSD of Different QM Methods for Chlorine and Bromine Interactions^b^

method	basis set	mean ΔΔ*E* to CCSD(T)/CBS	abs mean ΔΔ*E* to CCSD(T)/CBS	RMSD to CCSD(T)/CBS	mean runtime (CPUh)
Chlorine					
MP2	TZVPP	–0.68	0.68	1.06	1.01
	TZVPP+BSSE	0.35	0.35	0.51	3.23
M06–2X (-D3)	TZVPP	0.84	0.85	1.20	0.49
CCSD(T)	CBS	^a^	^a^	^a^	79.28
Bromine					
MP2	TZVPP	–0.79	0.79	1.30	1.15
	TZVPP+BSSE	0.32	0.33	0.50	3.56
M06–2X (-D3)	TZVPP	1.35	1.35	1.86	0.57
CCSD(T)	CBS	^a^	^a^	^a^	105.37

a No values reported, since CCSD­(T)/CBS is the reference for all other methods.

b Energy values are given as difference between corresponding method values and the reference calculations of CCSD­(T)/CBS in kJ/mol. Runtime is given in CPU hours.

## Conclusions

In this work, we investigated the potential of different QM methods to correctly assess halogen−π interactions with a focus on iodine. Adduct formation energy differences, ΔΔ*E*, between QM methods and reference calculations using CCSD­(T)/CBS, as well as the average runtime of single-point calculations, were reported. Results show that MP2 with the reasonably large basis set TZVPP is an excellent choice and is in very good agreement with reference calculations while maintaining feasible computational demands. With this study, we aim to provide a solid basis for characterizing halogen−π interactions in ab initio approaches and beyond. Similar to our previous experience,^27,28,30–32^ good performance of MP2/TZVPP appears to be transferable onto the evaluation of iodine−π systems. We were able to demonstrate that MP2/TZVPP remains a very good choice for chlorine and bromine interactions with the π-surface of benzene as well. It is interesting to note that applying a counterpoise correction enhances the accuracy of MP2/TZVPP for chlorine and bromine. However, the still very high computational demands make this method quite impractical for large data applications. Based on a good balance of accuracy and speed for MP2/TZVPP, this method will be employed to generate large data sets as a source for machine learning of this pharmaceutically interesting interaction. With such a QM-AI approach, high-accuracy interaction energies could become available on the millisecond scale.

## Computational Methods

### Structure Optimization

Geometry optimizations of the individual ligand model system (iodobenzene, chlorobenzene, and bromobenzene) and the amino acid model system of phenylalanine (benzene) were done at the MP2 level of theory using TURBOMOLE 7.7.1 ^72^ with a triple-ζ basis set (def2-TZVPP). Calculations were performed in combination with the resolution of identity (RI) technique and the frozen core approximation. Frozen core orbitals were defined using default settings, where orbitals with energies below −3.0 au are considered core orbitals. SCF convergence criterion was increased to 10^–8^ hartree. Relativistic effects for iodine were considered by an effective core potential (ECP).^73–81^

### Generation of Interaction Geometries

Interaction geometries of iodobenzene in complex with benzene were generated. Iodobenzenes were placed on a regular grid using *X*- and *Z*-translations (Figure 3a) for five different distances along the *Y*-axis (Figure 3b). Following previous approaches, an optimal σ-hole angle of α_C–I···π‑plane_ = 180° was used. In this angle definition (α_C–I···π‑plane_), the respective point on the π-plane is individually determined by the normal to the plane through the iodine atom. Due to the symmetric nature of benzene, only one quadrant of the grid was considered. With this procedure, a total of 495 interaction geometries were generated to carry out a single-point calculation. For the comparison to CCSD­(T)/CBS reference calculations, the same smaller subsets (∼30% of all geometries) were used for all halobenzenes.

**Figure 3:**
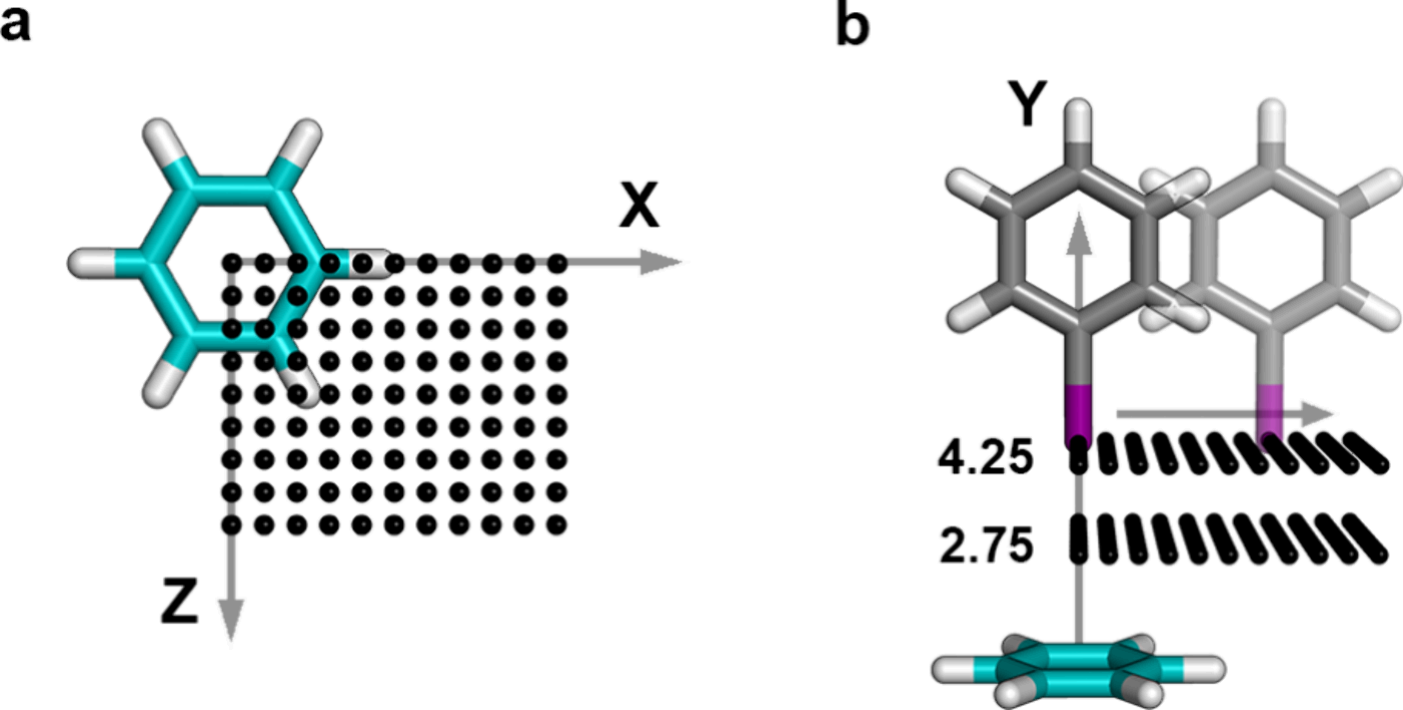
Overview of the interaction geometry generation on a regular grid. (a) Grid points on the *XZ*-plane were generated with dimensions *X*_translation_ = [0.0–5.0 Å], *Z*_translation_ = [0.0–4.0 Å] in steps of 0.5 Å. (b) Grid points were generated for five different distances *d*_I···π‑plane_ between the halogen atom and the benzene plane, *d*_I···π‑plane_ = [2.75 3.25, 3.5, 3.75, and 4.25 Å]. In this distance definition (*d*_I···π‑plane_) the respective point on the π-plane is individually determined by the normalization to the plane through the iodine atom. Figures were prepared with PyMOL.^82^

### QM Methods, Basis Sets, and Adduct Formation Energies

An overview of the different methods and basis set combinations can be seen in Table 1. All single-point calculations were carried out using TURBOMOLE 7.7.1 on the JUSTUS2–bwHPC Cluster,^83^ where a standard node has a 2 × Intel Xeon E6252 Gold (Cascade Lake) CPU (2.1 GHz base, 3.7 GHz max. accelerated) with 192GB or 384GB memory. Calculations were done in combination with the resolution of identity (RI) technique and the frozen core approximation, if applicable. Frozen core orbitals were defined using default settings, where orbitals with energies below −3.0 au are considered core orbitals. SCF convergence criterion was increased to 10^–8^ hartree. Relativistic effects for iodine were considered by an effective core potential (ECP). Methods of choice comprise MP2, MP3, SCS-MP2, B3LYP, M06–2X, and TPSS. For selected methods and basis set combinations (see Table 1), energy values were counterpoise corrected using the procedure of Boys and Bernardi to eliminate basis set superposition errors (BSSEs). Basis sets included in the study were the triple-ζ basis set def2-TZVPP and the diffuse function enhanced variant def2-TZVPPD. Further, the correlation consistent basis sets cc-pVNZ-PP and the augmented basis sets aug-cc-pVNZ-PP (*N* = *T*, *Q*) were used with an additional pseudo potential for iodine (denoted by the “-PP” suffix). The DFT functionals TPSS, B3LYP, and M06–2X were augmented with an empirical dispersion correction as proposed by Grimme et al.,^61^ which is indicated by adding “(-D3)” to the name. For the previously investigated chlorobenzene and bromobenzene, a small set of methods and basis sets was applied to provide the possibility of comparing our data for iodine to both less heavy halogens.

As a reference, single-point calculations at the complete basis set limit approximation were carried out using an extrapolation scheme proposed by Halkier et al.^65^ Higher-order correlation energy was calculated using the following equation:

$$\Delta E_{\mathrm{CBS}}^{\mathrm{CCSD}(T)}=\Delta E_{\mathrm{CBS}}^{\mathrm{MP}2}+(\Delta E^{\mathrm{CCSD}(T)}-\Delta E^{\mathrm{MP}2}{)}_{\mathrm{cc}-\mathrm{pVTZ}-\mathrm{PP}}$$ 1

This is due to the assumption that the difference between the CCSD­(T) and MP2 interaction energies depends only slightly on the basis set and can therefore be estimated using a small or medium basis set, such as cc-pVTZ-PP. Δ*E*_CBS_^MP2^ represents the energy at the complete basis set limit and can be determined as follows:

$$\Delta E_{\mathrm{CBS}}^{\mathrm{MP}2}=\frac{\Delta E_{X}^{\mathrm{MP}2}X^{3}-\Delta E_{Y}^{\mathrm{MP}2}Y^{3}}{X^{3}-Y^{3}}$$ 2

where *X* and *Y* denote the cardinal numbers of the cc-pVTZ-PP and cc-pVQZ-PP basis set (*T* = 3 and *Q* = 4). Adduct formation energies were calculated as

$$\Delta E=(E_{\mathrm{complex}}-(E_{\mathrm{halobenzene}}+E_{\mathrm{benzene}}))$$ 3

and reported as kJ/mol.

## Supplementary Material





## Data Availability

PyMOL is an open-source software maintained and distributed by Schrödinger. There is an open-source version of PyMOL available at: https://github.com/schrodinger/pymol-open-source. Python and all of its’ packages is an open-source programming language available and downloadable from https://www.python.org/. Detailed results of the QM method comparison and an overview of the individual data points are provided in spreadsheet format (xlsx) as Supporting Information. TURBOMOLE is a purchasable software maintained and distributed by the *TURBOMOLE GmbH*. Demo versions are available at https://www.turbomole.org/. The licensed software was provided to us by the bwHPC Cluster JUSTUS2.

## Notes

Additional details and figures of the energy evaluation and visualization (PDF)
Detailed table of the individual data points with corresponding adduct formation energies and differences to the reference (XLSX)

## Abbreviations

XB: halogen bond
X: halogen
QM: quantum mechanical
TPSS, Tao: Perdew, Staroverov, and Scuseria exchange functional
D3: dispersion correction type 3
MP2: Mo̷ller–Plesset perturbation method order 2
TZVPP: valence triple-ζ with two sets of polarization functions
TZVPPD: valence triple-ζ with two sets of polarization functions and diffuse function
BSSE: basis set superposition error
cc-pVTZ-PP: correlation-consistent polarized valence triple-ζ function with pseudopotentials
cc-pVQZ-PP: correlation consistent polarized valence quadruple-ζ function with pseudopotentials
B3LYP: Becke, 3-parameter, Lee–Yang–Parr functional
M06–2X: Minnesota 06 hybrid functional with double Hartree–Fock exchange
CCSD­(T): coupled cluster with single, double, and perturbative triple excitations
CBS: complete basis set
SCF: self-consistent field
RI: resolution of identity
DFT: density functional theory
ECP: effective core potential
RMSD: root-mean-square deviation
GGA: Generalized Gradient Approximation
SCS-MP2: spin-component-scaled MP2
