# Supplementary material for: Comparison of QM Methods for the Evaluation of Halogen−π Interactions for Large-Scale Data Generation
Source: J Chem Theory Comput. 2025 Jun 9;21(12):6174–83. doi: 10.1021/acs.jctc.5c00456 (PMC12199455; doi:10.1021/acs.jctc.5c00456)
Supplement: Supplementary file 1 [file ct5c00456_si_001.pdf]

Supporting Information

Comparison of QM Methods for the Evaluation of Halogen-π Interactions for Large Scale Data Generation

Marc U. Engelhardt[1], Markus O. Zimmermann[1,2], Finn Mier[1], Frank M. Boeckler[1,2,]*

[1] Laboratory for Molecular Design & Pharmaceutical Biophysics, Institute of Pharmaceutical Sciences, Department of Pharmacy and Biochemistry, Eberhard Karls Universität Tübingen, 72076 Tübingen, Germany.

[2] Interfaculty Institute for Biomedical Informatics (IBMI), Eberhard Karls Universität Tübingen, 72076 Tübingen, Germany.

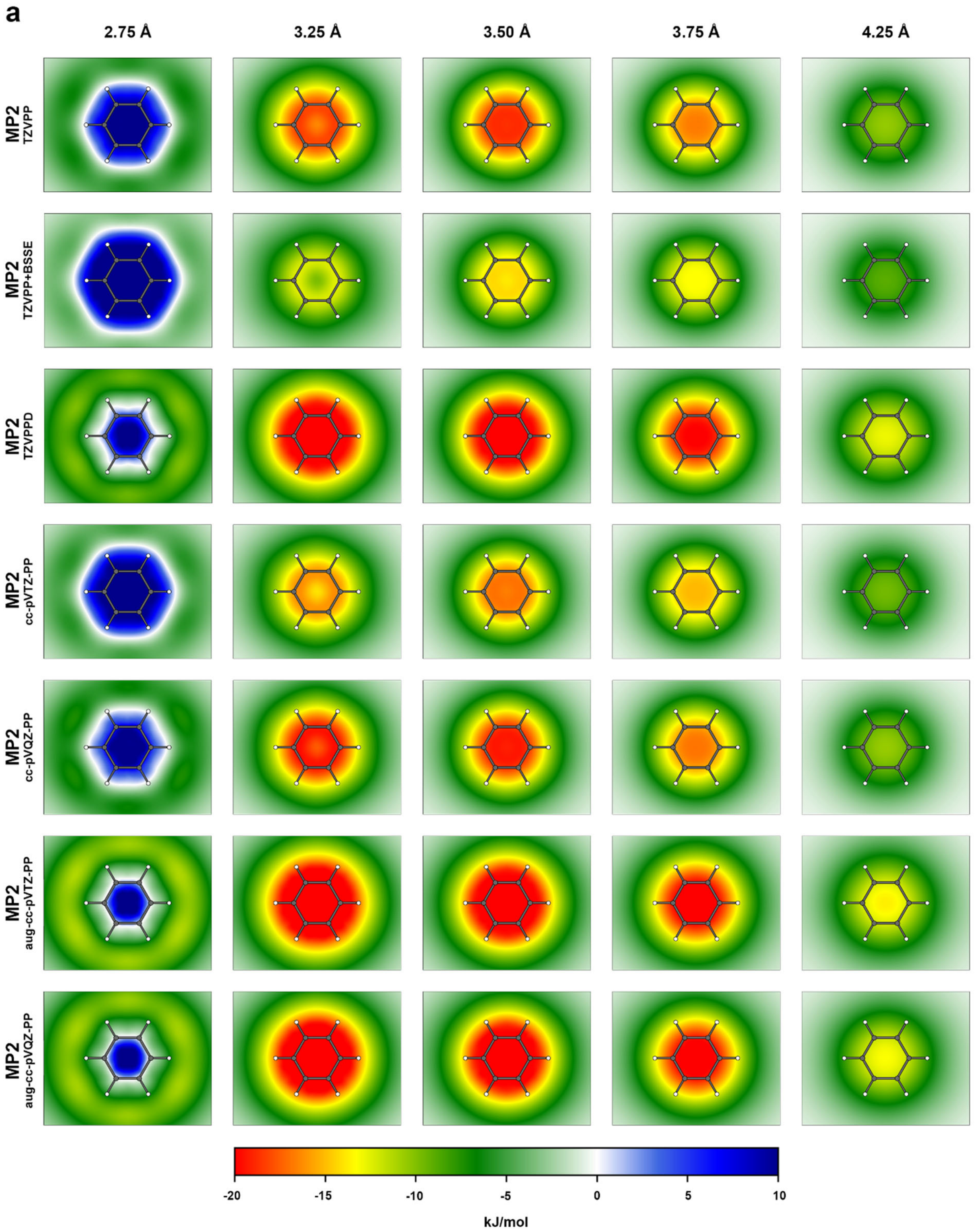

a
2.75 Å
3.25 Å
3.50 Å
3.75 Å
4.25 Å
MP2 TZVPP
MP2 TZVPP+BSSE
MP2 TZVPPD
MP2 cc-pVTZ-PP
MP2 cc-pVQZ-PP
MP2 aug-cc-pVTZ-PP
MP2 aug-cc-pVQZ-PP
-20
-15
-10
-5
0
5
10
kJ/mol

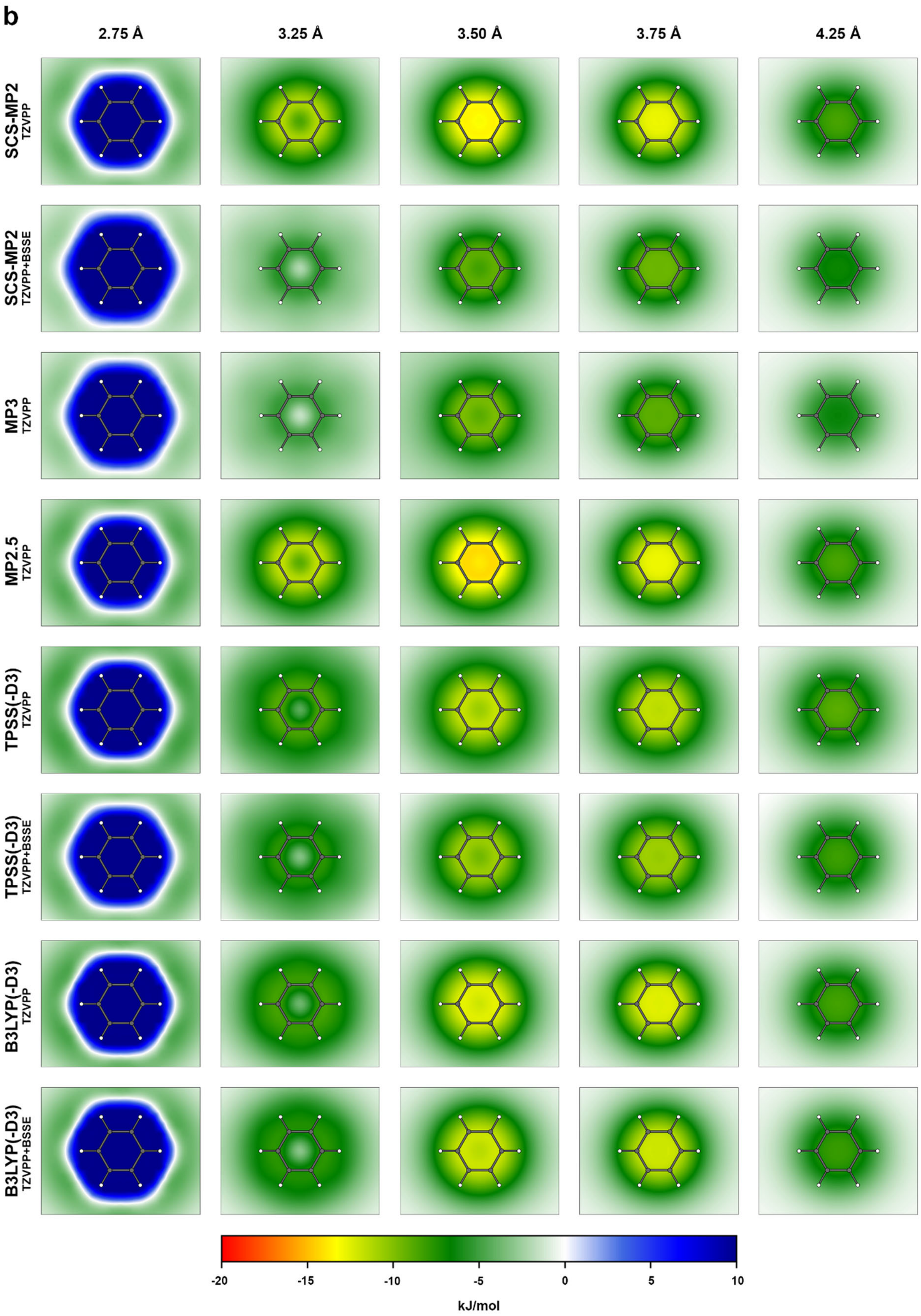
b
2.75 Å
3.25 Å
3.50 Å
3.75 Å
4.25 Å
SCS-MP2
TZVPP
SCS-MP2
TZVPP+BSSE
MP3
TZVPP
MP2.5
TZVPP
TPSS(-D3)
TZVPP
TPSS(-D3)
TZVPP+BSSE
B3LYP(-D3)
TZVPP
B3LYP(-D3)
TZVPP+BSSE
-20
-15
-10
-5
0
5
10
kJ/mol

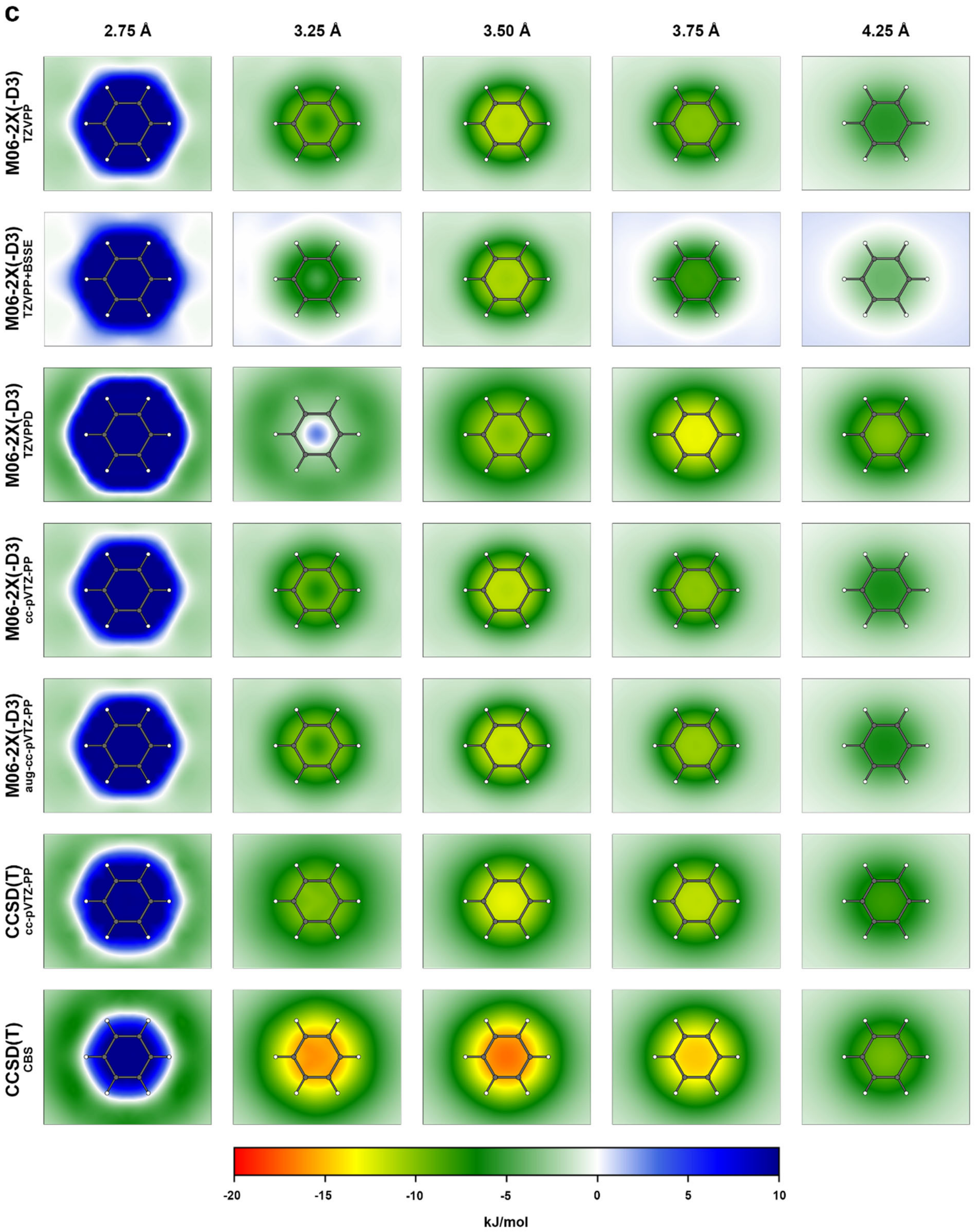

Figure S1. Iodine adduct formation energy surfaces of all evaluated method and basis set combinations. Surfaces represent the halogen-π interaction energies ΔE of iodobenzene in

complex with the targeted benzene at distances of $d_{I\cdots\pi\text{-plane}}$ = [2.75 Å, 3.25 Å, 3.5 Å, 3.75 Å, 4.25 Å]. The iodobenzene is oriented perpendicular to the π-plane. Data points of the surface are interpolated and colored according to the given energy scale. Positive energies and negative energies are capped to 10 kJ/mol and -20 kJ/mol, respectively, for better visibility. (a) Energy surface of all MP2 variants. (b) Energy surfaces of SCS-MP2, MP3, MP2.5, TPSS(-D3), and B3LYP(-D3) calculations. (c) Energy surfaces of M06-2X(-D3) and CCSD(T) calculations. Figures were prepared using custom Python scripts and the *matplotlib* library.

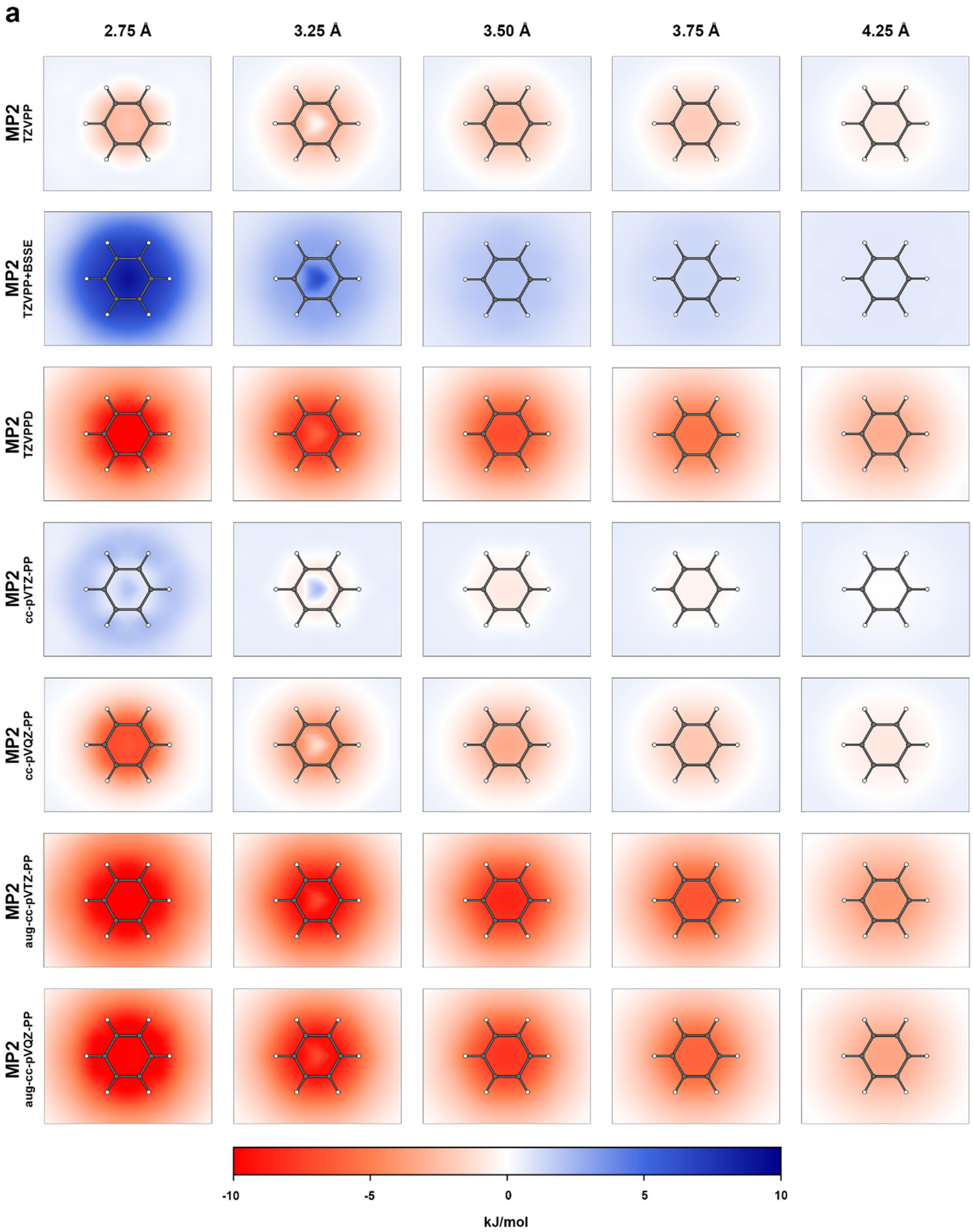
a
2.75 Å
3.25 Å
3.50 Å
3.75 Å
4.25 Å
MP2 TZVPP
MP2 TZVPP+BSSE
MP2 TZVPPD
MP2 cc-pVTZ-PP
MP2 cc-pVQZ-PP
MP2 aug-cc-pVTZ-PP
MP2 aug-cc-pVQZ-PP
-10
-5
0
5
10
kJ/mol

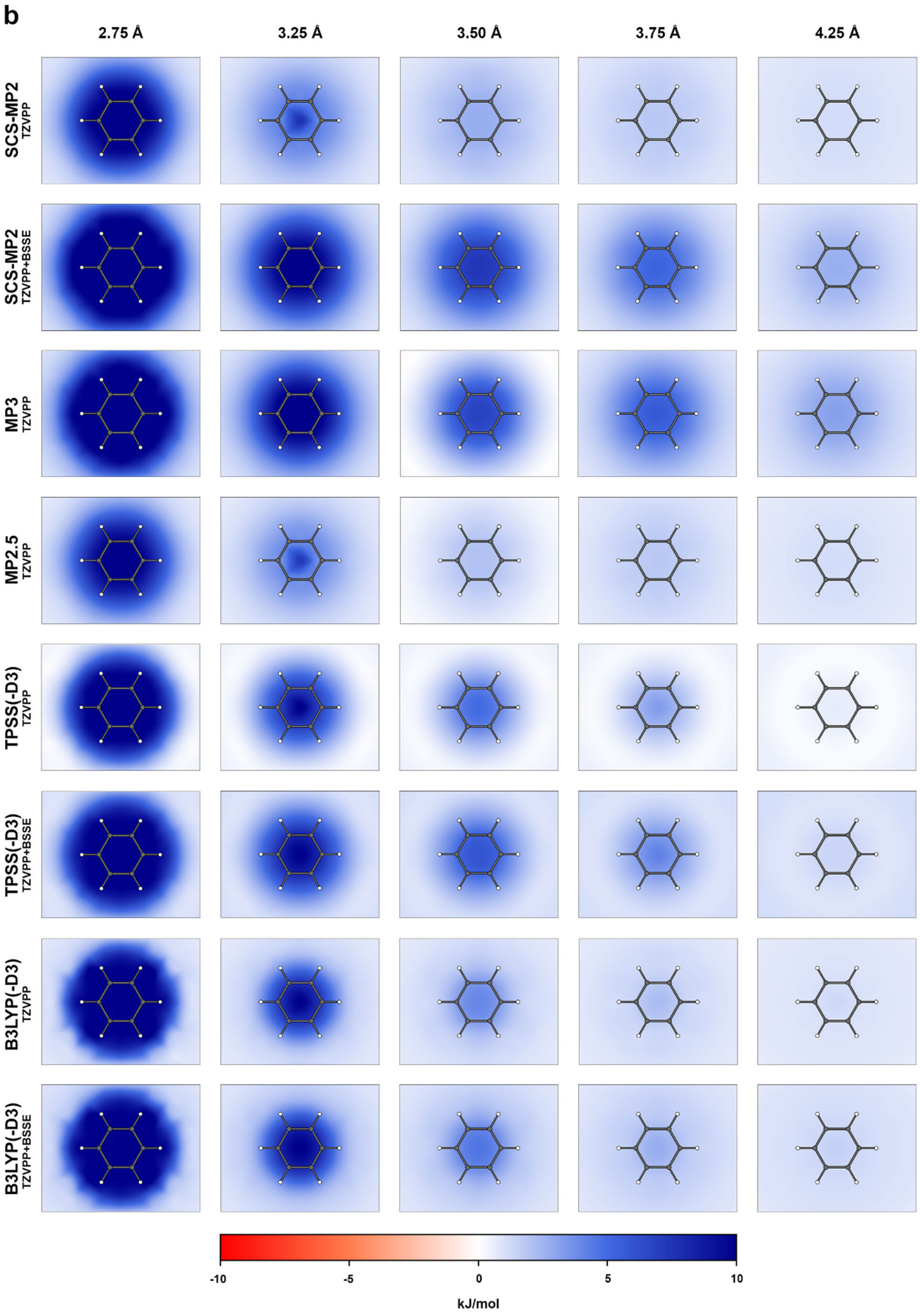
b
2.75 Å
3.25 Å
3.50 Å
3.75 Å
4.25 Å
SCS-MP2 TZVPP
SCS-MP2 TZVPP+BSSE
MP3 TZVPP
MP2.5 TZVPP
TPSS(-D3) TZVPP
TPSS(-D3) TZVPP+BSSE
B3LYP(-D3) TZVPP
B3LYP(-D3) TZVPP+BSSE
-10
-5
0
5
10
kJ/mol

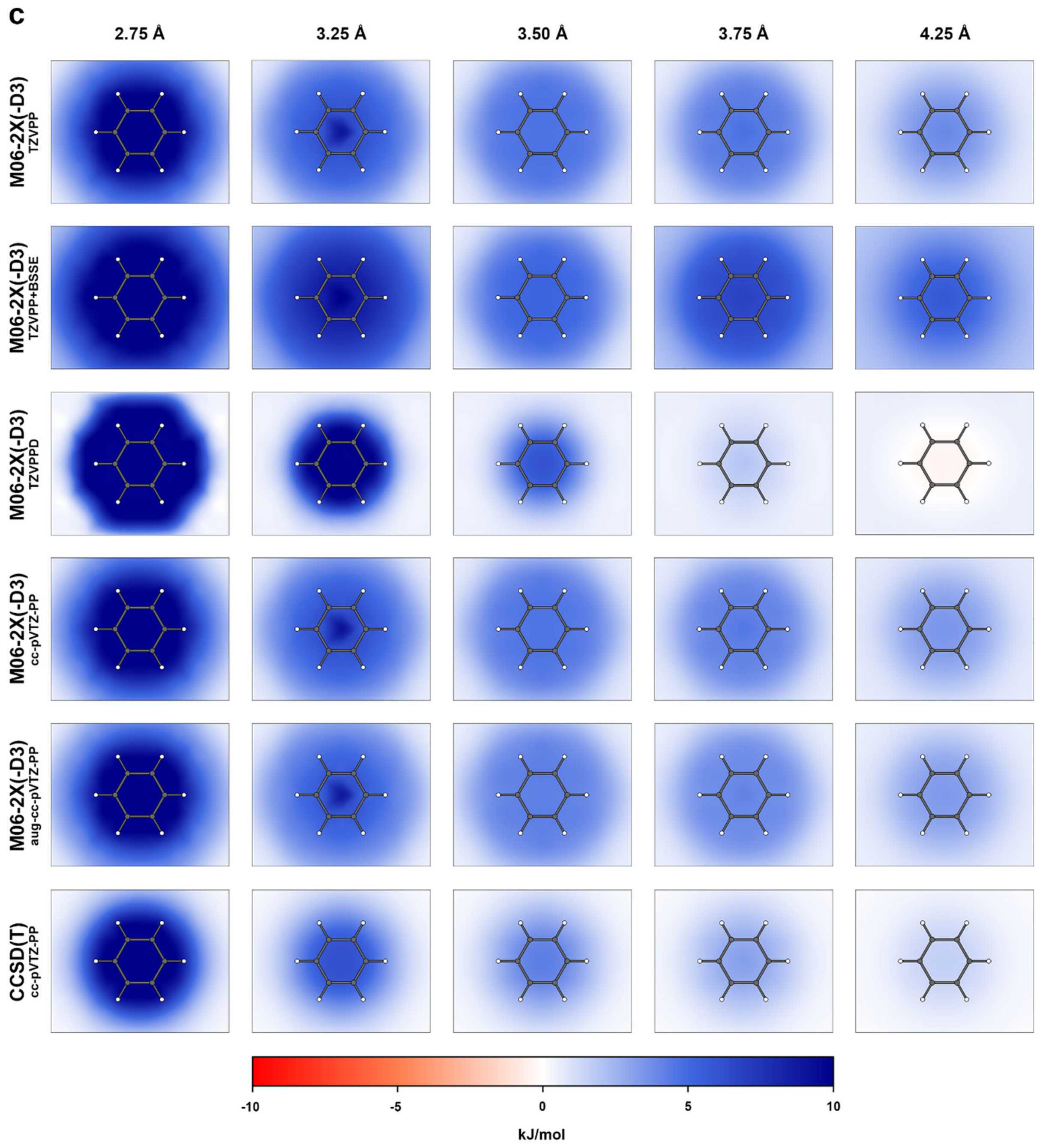

Figure S2. Iodine energy surfaces of the difference in adduct formation energies ΔΔE of all evaluated method and basis set combinations to the reference CCSD(T)/CBS. Surfaces represent the halogen-π interaction energy differences ΔΔE of iodobenzene in complex with the targeted benzene at distances of $d_{I\cdots\pi\text{-plane}}$ = [2.75 Å, 3.25 Å, 3.5 Å, 3.75 Å, 4.25 Å]. The iodobenzene is oriented perpendicular to the π-plane. Data points of the surface are interpolated and colored

according to the given energy scale. Positive energies and negative energies are capped to 10 kJ/mol and -10 kJ/mol, respectively, for better visibility. (a) Surfaces of all MP2 variants. (b) Surfaces of SCS-MP2, MP3, MP2.5, TPSS(-D3), and B3LYP(-D3) calculations. (c) Surfaces of M06-2X(-D3) and CCSD(T) calculations. Figures were prepared using custom Python scripts and the *matplotlib* library.

Chlorine

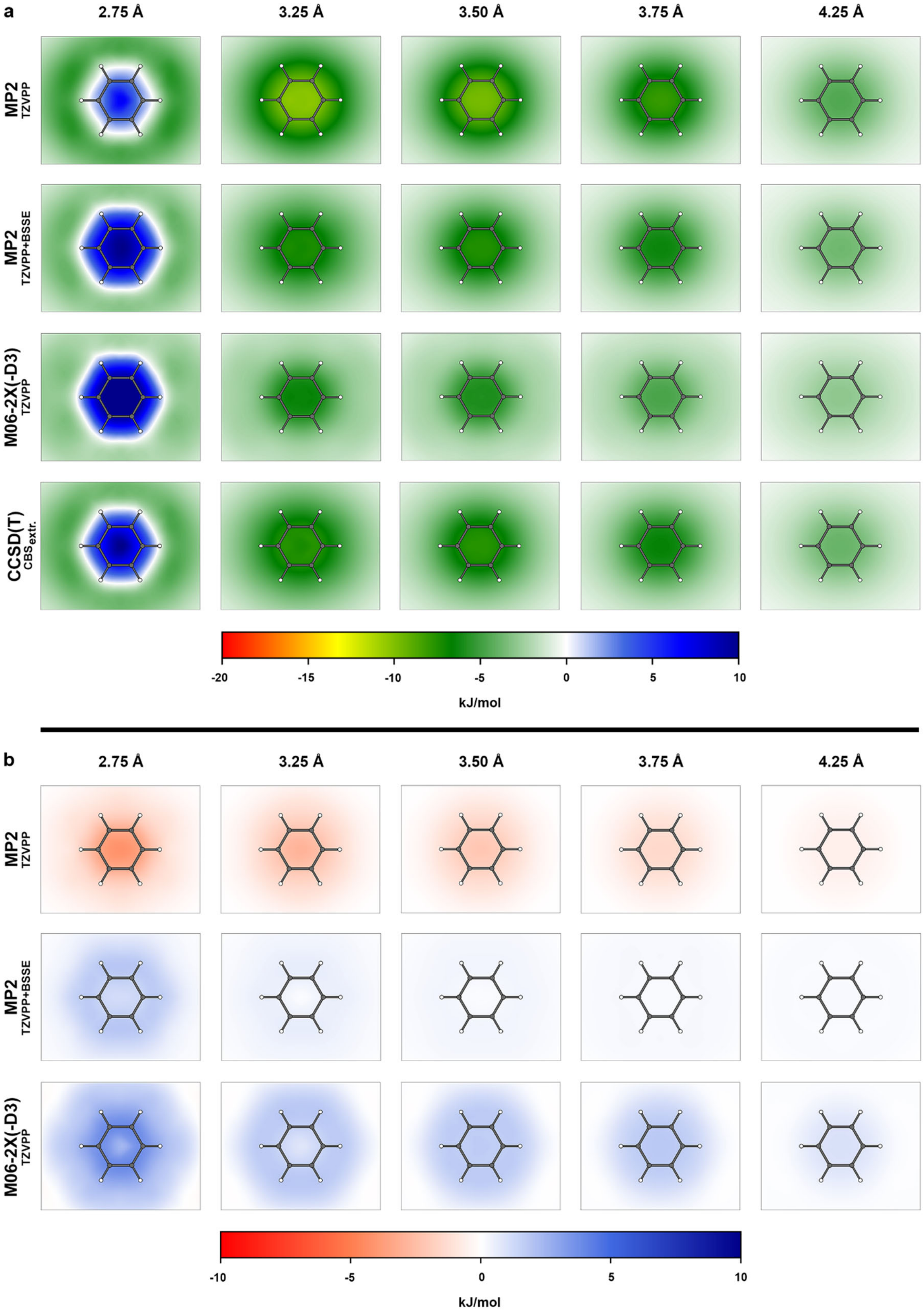
a
2.75 Å
3.25 Å
3.50 Å
3.75 Å
4.25 Å
MP2 TZVPP
MP2 TZVPP+BSSE
M06-2X(-D3) TZVPP
CCSD(T) CBSextr.
-20
-15
-10
-5
0
5
10
kJ/mol
b
2.75 Å
3.25 Å
3.50 Å
3.75 Å
4.25 Å
MP2 TZVPP
MP2 TZVPP+BSSE
M06-2X(-D3) TZVPP
-10
-5
0
5
10
kJ/mol

Figure S3. Chlorine adduct formation energy surfaces of evaluated method and basis set combinations, as well as energy difference surfaces between each method and CCSD(T)/CBS. Surfaces represent the halogen-π interaction energies ΔE of chlorobenzene in complex with the targeted benzene at distances of $d_{Cl\cdots\pi\text{-plane}}$ = [2.75 Å, 3.25 Å, 3.50 Å, 3.75 Å, 4.25 Å]. The chlorobenzene is oriented perpendicular to the π-plane. Data points of the surface are interpolated and colored according to the given energy scale. (a) Surfaces of adduct formation energies ΔE. Positive energies and negative energies are capped to 10 kJ/mol and -20 kJ/mol, respectively. (b) Surfaces of the difference between adduct formation energies of evaluated methods and the reference CCSD(T)/CBS (calculated as $\Delta\Delta E = \Delta E_{method} - \Delta E_{CCSD(T)/CBS}$). Positive and negative differences were capped to 10 kJ/mol and -10 kJ/mol. Figures were prepared using custom Python scripts and the *matplotlib* library.

Bromine

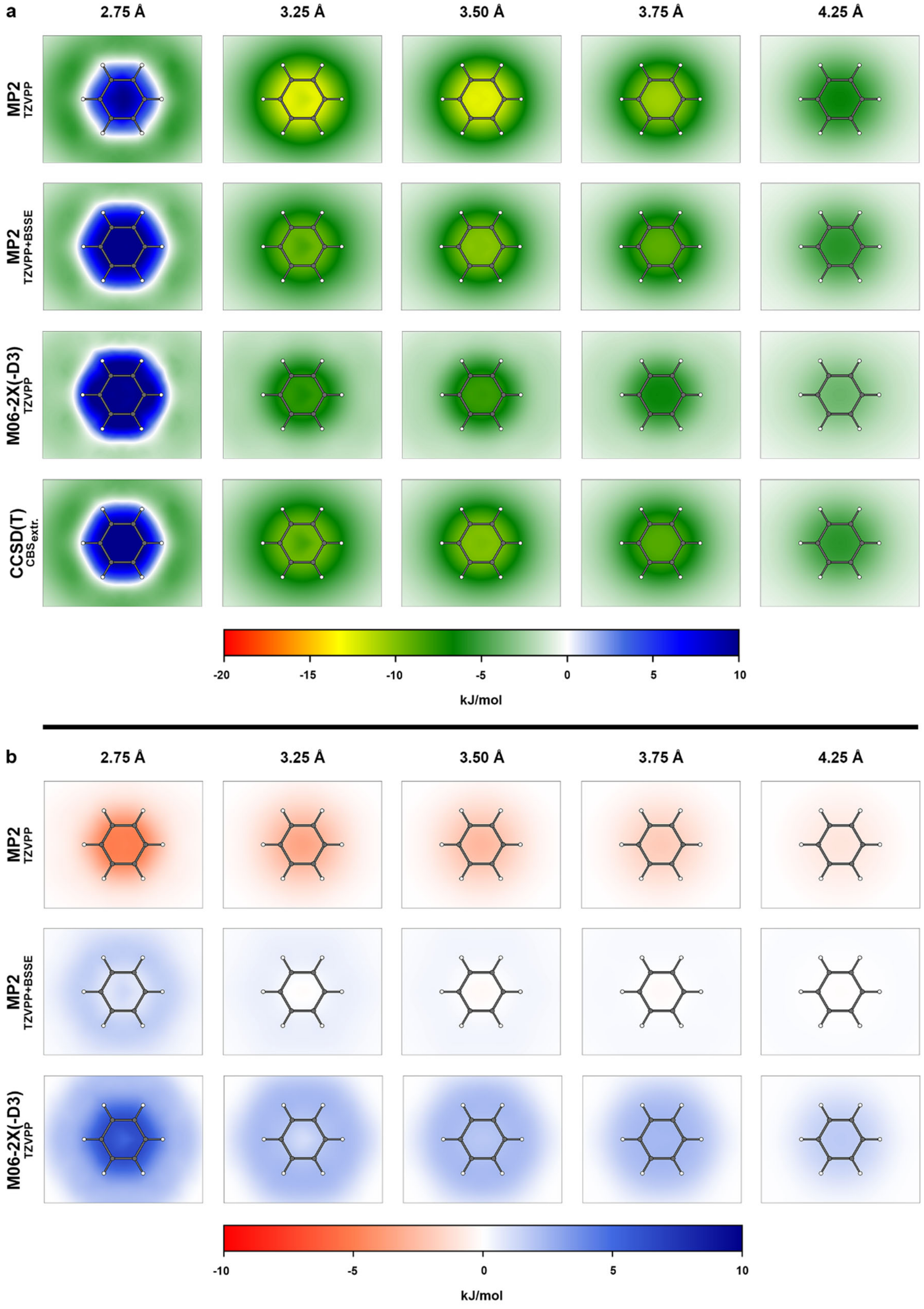
a
2.75 Å
3.25 Å
3.50 Å
3.75 Å
4.25 Å
MP2 TZVPP
MP2 TZVPP+BSSE
M06-2X(-D3) TZVPP
CCSD(T) CBSextr.
-20
-15
-10
-5
0
5
10
kJ/mol
b
2.75 Å
3.25 Å
3.50 Å
3.75 Å
4.25 Å
MP2 TZVPP
MP2 TZVPP+BSSE
M06-2X(-D3) TZVPP
-10
-5
0
5
10
kJ/mol

Figure S4. Bromine adduct formation energy surfaces of evaluated method and basis set combinations, as well as energy difference surfaces between each method and CCSD(T)/CBS. Surfaces represent the halogen-π interaction energies ΔE of bromobenzene in complex with the targeted benzene at distances of $d_{Br\cdots\pi\text{-plane}}$ = [2.75 Å, 3.25 Å, 3.50 Å, 3.75 Å, 4.25 Å]. The bromobenzene is oriented perpendicular to the π-plane. Data points of the surface are interpolated and colored according to the given energy scale. (a) Surfaces of adduct formation energies ΔE. Positive energies and negative energies are capped to 10 kJ/mol and -20 kJ/mol, respectively. (b) Surfaces of the difference between adduct formation energies of evaluated methods and the reference CCSD(T)/CBS (calculated as $\Delta\Delta E = \Delta E_{method} - \Delta E_{CCSD(T)/CBS}$). Positive and negative differences were capped to 10 kJ/mol and -10 kJ/mol. Figures were prepared using custom Python scripts and the *matplotlib* library.
